# Thinking outside of the [cold] box: implementing a human-centered design approach to understand barriers and craft solutions to cold chain equipment maintenance in Niger

**DOI:** 10.1186/s40545-023-00654-w

**Published:** 2023-11-16

**Authors:** Wendy Prosser, Nicole Danfakha, Brittany Thurston, Aminta Gueye, Samira Matan, Cheikh Tidiane, Emily Kitts, Ibrahim Alkassoum, Yannick Agui, Abdoulaye Brah-Bouzou, Harouna Dembo, Mata Garba

**Affiliations:** 1 JSI, Boston, USA; 2 ThinkPlace/Senegal, Dakar, Senegal; 3 JSI/Niger, Niamey, Niger; 4 Ministry of Health/Niger, Niamey, Niger; 5 ThinkPlace, ThinkPlace, London, UK; 6 Research Consultant, Research Consultant, Niamey, Niger

**Keywords:** Vaccine, Immunization, Cold chain equipment, Maintenance, Human-centered design, Supply chain

## Abstract

**Background:**

Vaccines require cold chain equipment (CCE) to ensure quality and potency, yet the risk of CCE failing is well-documented, often due to lack of equipment maintenance. While general barriers to a reliable CCE maintenance system are known, little has been done to understand the barriers from the cold chain technician’s perspective. This human-centered design (HCD) study in Niger sought to better understand the gap in the current maintenance approaches from the technicians’ perspectives and to collaboratively identify forward-thinking solutions.

**Methods:**

The research team collected data through semi-structured and open-ended in-depth consultations. Rapid qualitative research was followed by co-creation workshops with study participants to identify solutions.

**Results:**

The research team conducted 20 in-depth consultations in two regions with participants directly involved in the management of the cold chain. Fourteen people participated in the online co-creation workshop, and 20 people participated in the in-person workshop. Insights were organized in three main areas: (1) the lack of system agility and ability to optimize resources constrain performance of the maintenance system; (2) cold chain is often an afterthought within the overall context of immunization services and should instead be prioritized; and (3) knowledge sharing across the system and key stakeholders is ad hoc with limited understanding of roles and responsibilities. During the co-creation workshop, participants identified four main concepts as potential solutions: (1) create an on-line platform to connect people and to share knowledge across regions; (2) develop practical CCE technical resources; (3) use gamification and friendly competition to motivate staff to value maintenance; and (4) create a real-time maintenance tracking system.

**Discussion:**

This study took a unique HCD approach to engage people directly involved in CCE maintenance to more deeply understand challenges with the current system and create space to identify innovative solutions that are tailored to the context. The results demonstrate that stakeholders can identify potential solutions that have not been part of the typical approaches to a maintenance system. This HCD approach has implications for all global health challenges and demonstrates a methodology that can encourage key stakeholders to think about problems and solutions differently.

## Background

Immunization has been proven to be a cost-effective intervention to save lives and reduce morbidity [1]. One of the unique characteristics of vaccines is the requirement to be stored in cold chain equipment (CCE) throughout the supply chain, from manufacturer to point of administration at a health facility, to ensure quality and potency of the vaccines [2]. The risk of temperature excursions outside of the ideal temperature range while being stored in CCE or during transportation and distribution in low- and middle-income countries (LMIC) is well-documented. Improving the performance of the CCE is becoming a priority for immunization programs, partners, and donors [3, 4].

In recent years, improved design in new CCE models increased the ability of CCE to maintain its ideal temperature range, and significant investments have been made in new CCE for LMICs through Gavi, the Vaccine Alliance and the Cold Chain Equipment Optimization Platform, as well as additional equipment procured by Gavi and other donors to support the introduction of the COVID-19 vaccine [5].

Where investment falls short, however, is in the maintenance of the CCE. To ensure functionality and a long lifespan of the equipment, regular and documented maintenance is essential. Preventive maintenance involves regularly cleaning the equipment, defrosting as necessary, tracking the temperature of the equipment twice daily, and responding to any temperature excursions. At a health facility, preventive maintenance is typically implemented by health workers involved in the immunization program; at higher level storage points, it is typically conducted by immunization program or logistics staff. Corrective maintenance is performed when the equipment fails, shows signs of failure, or requires replacing parts, typically done by a trained cold chain technician.

Government cold chain maintenance systems are not keeping pace with the growing need of CCE as demonstrated by low scores on preventive maintenance during country assessments [6]. General challenges to maintenance systems in LMICs include lack of funds to support CCE technicians to do their jobs, lack of spare parts, little visibility into the true need of maintenance, and insufficient training for technicians [7]. The need for stronger maintenance systems is well-known and has been highlighted by immunization programs and partners, including as a priority in Gavi’s Immunization Supply Chain Strategy [8–11].

Maintenance systems have evolved and changed over the years to respond to entrenched obstacles. Alternative approaches include outsourcing maintenance to a private company [12] or using a service-bundled contract where manufacturers are required to install new equipment and respond to maintenance needs while it is under warranty [13]. While these approaches solve some of the issues related to maintenance, they still fall short of a sustainable, reliable, and responsive maintenance system.

While general barriers to a reliable maintenance system are known, little has been done to understand the drivers of the barriers from the cold chain technician’s perspective and to engage technicians in thinking creatively about potential solutions to the barriers they face. In Niger, more than 1,100 health facilities provide immunization services, supported by more than 2,000 pieces of CCE, including large walk-in cold rooms for regional and national storage sites, equipment that runs on the electricity grid, and solar powered equipment. The immunization program employs cold chain technicians to maintain the equipment yet recognizes the maintenance system is underperforming. The program has recently contracted a private provider to outsource maintenance for larger equipment and higher volume sites at the regional level in an effort to address the gaps in the maintenance system. This study was designed to better understand the gap in the current maintenance approaches from the perspective of the technicians and to collaboratively identify potential forward-thinking solutions in an effort to improve the maintenance system.

## Methods

### Study objectives and design

This study applied a human-centered design (HCD) approach in an attempt to understand the problems directly from the people facing them, build deep empathy, and generate possible ideas and new solutions to address their challenges and meet their specific needs [14–16]. The study engaged and supported the teams directly involved in cold chain maintenance to reconceptualize the barriers and root causes of an unresponsive maintenance system and to co-create context-appropriate solutions. The study had two objectives: (1) Understand the entrenched obstacles that limit a reliable and functioning cold chain maintenance system and (2) to co-design a forward-thinking managed CCE maintenance system. The study was designed to gain insight into a few of the areas that are critical to a well-functioning CCE maintenance system, including system management and procedures, resources for execution, and temperature monitoring practices. This qualitative study used HCD approaches to ensure the engagement and collaboration of stakeholders at all levels of the health system.

### Study setting and sampling criteria

The study was implemented in Niger in two regions, Tahoua and Maradi, with data collection conducted in February and March, 2023. The co-creation workshop brought participants together in April 2023. The research team included HCD experts, qualitative researchers and project staff with expertise in immunization supply chain. The selection of regions was done based on purposeful sampling with alignment from the national immunization manager and supply chain team. As a qualitative study, sample size calculations were not used; instead, the study aimed to consult enough respondents to achieve data saturation on the research questions. Within each region, two districts and two health facilities were selected to ensure perspectives of stakeholders directly involved in managing and maintaining CCE or making decisions related to maintenance.

### Data collection, analysis and co-creation

Two local researchers were trained by the HCD experts on interview techniques and the semi-structured consultation guides. The research team conducted one-on-one consultations using the guides. The consultations were approximately one hour long. Consultations were conducted with cold chain technicians, representatives from the government entity providing maintenance, regional and district immunization coordinators, financial managers at region and district levels, and healthcare workers directly involved in administering vaccines at health facilities. These respondents were selected based on their involvement in managing and maintaining CCE, such as through daily interactions, in the case of facility-based healthcare workers, or through prioritizing maintenance, in the case of financial managers.

Data collection was conducted in two phases. The first phase was conducted in Tahoua and led by the full research team, made up of HCD experts together with the local research team. The second phase of data collection was conducted in Maradi and was led by the local research team with support from the HCD experts provided remotely. The research team took notes during the consultations and summarized the key findings of each consultation. The research team de-briefed daily to discuss emerging insights and discuss areas to probe further on during subsequent consultations.

Upon completion of data collection, the research team came together to begin reviewing and processing the information gathered during consultations. The team used the “insight generation” technique to analyze results and began organizing the data and key findings into emerging themes. These themes were then further synthesized and transformed into opportunity areas. The insights were translated into “How Might We” questions which reframed the insights into opportunity areas for design during the co-creation workshop. The questions were designed to be broad enough to not limit solutions but narrow enough to keep the solutions focused on addressing the barrier at hand. Additionally, user personas and a journey map were developed from the consultations findings. This step helped to gain insight into the underlying challenges with the CCE maintenance system. The research team developed “personas”—short stories and descriptions centered around key stakeholders’ experiences based on the synthesized data—to illustrate selected challenges during the co-creation workshop. Using personas also ensured confidentiality of the research participants.

The co-creation process was conducted through two co-creation workshops and one validation meeting. The first co-creation workshop took place online during a half-day session with global stakeholders and thought leaders in cold chain and immunization supply chain. The purpose of this initial workshop was to identify potential solutions that could be used to spark ideas during the in-person workshop. The second co-creation workshop was conducted in person in Niamey during a two-day session with respondents who were consulted during data collection. The purpose of this second co-creation workshop was to validate emerging findings from the data collection phase, strengthen the understanding of barriers to CCE maintenance, and identify locally relevant interventions to address the challenges. Ideas generated during the first co-design workshop were used as an inspiration and incorporated into new ideas generated during the second co-design workshop. The How Might We questions were used for brainstorming during the workshops. Ideas were fleshed out to include detailing how the idea will work, who would be involved, how the idea differed from others, and the problem the idea addressed. A half-day validation session included national level decision makers, partners, and donors to begin socializing the insights and ideas generated during the consultations and co-design workshops. Participants were invited to share additional insights and ideas during the validation meeting.

Ethical approval was obtained from JSI’s corporate Institutional Review Board committee, which approved the study protocol based on recognition that it does not meet the definition of human subjects research. The research team obtained participation consent prior to any observation or engagement. Research team members read the consent form to participants and provided them time to ask questions and clarify consent. As described in the consent form provided to the participants, all participants could request to withdraw from participation at any time.

## Results

The research team conducted 20 in-depth consultations in two regions with participants who are directly involved in the management of the cold chain, including maintenance, making financial decisions related to maintenance, or direct users of the cold chain (Table 1). After data collection, the research team led data synthesis and insight identification to serve as a starting point for the co-creation workshops. Fourteen people participated in the online co-creation workshop, and 20 people participated in the in-person workshop including five of the same participants from the online workshop (four researchers and one Niger-based project supply chain technical expert). Ten additional stakeholders participated in the validation meeting.

**Table 1:** Participants of in-depth consultations

Title of participant (French acronym)	Tahoua	Maradi
Versatile Maintenance Technician (TPM)	1	2
Maintenance and Repair Service for Operating Equipment (SERMEX, government entity providing maintenance)	1	1
Regional Immunization Coordinator (CRI, regional level)	1	1
Departmental Immunization Coordinator (CDI, district level)	2	2
Head of Financial Affairs Department	1	1
Financial manager (district level)	2	1
Health Facility In-Charge	2	2
TOTAL	10	10

Results of data collection and synthesis identified three emergent themes.

### Themes and insights

#### Theme 1: agility of the system and optimization of resources

The study showed that the cold chain maintenance system in Niger is based on a hierarchical organizational structure, which leads to internal communication challenges between various stakeholders, a lack of agility, and inefficient use of available resources. The research team identified three main insights under this theme.

While the government has a maintenance department and trained technicians, the system is highly dependent on partners for funding to support maintenance activities. According to the research, each year, the government immunization teams develop workplans, which include preventive and corrective maintenance, and submit the plans to partners such as UNICEF, Gavi and WHO to request financial and material support. The availability of funding is then tied to partners’ priorities and may not be available for much-needed maintenance activities. Dependence on external partners creates a system that is rigid, slow, and unable to respond quickly and efficiently to maintenance needs.

Communication between stakeholders involved in the CCE maintenance system lacks clarity and structure. The health system in Niger has a clear chain of command from national to regional and district levels; despite this clear organizational structure, communication related to the CCE is ad hoc and unstructured. Many participants expressed that when faced with problems they were often unsure who they should turn to. For example, versatile maintenance technicians (TPMs) report directly to the Maintenance and Repair Service for Operating Equipment (SERMEX), which should be the first point of contact when a TPM faces a maintenance problem that cannot be resolved. Yet, the consulted TPMs and SERMEX explained they were rarely in contact with each other.

Health workers at facilities are diligent about collecting cold chain temperature data and recording on paper temperature logs twice daily as per government policy. This data is then collated into a monthly report and kept on site; it is shared more broadly only if regional or national staff conduct supervision activities. Participants indicated that they do not have a clear understanding of the utility of cold chain temperature data for any management or long-term planning purpose. The data is collected because it is a daily task and required by government policy, yet the data is not used for broader planning decisions. Participants indicated that they react to the data that show significant temperature excursions by moving vaccines to a different CCE, yet the action taken is typically not documented, and it is not linked to a request for maintenance. The absence of a clear system or guidelines on how to effectively use temperature data contributes to the lack of optimization of available resources.

#### Theme 2: prioritizing cold chain

The findings indicated that the immunization program effectively de-prioritizes cold chain management and maintenance, as described in two insights.

Partners are an integral part of the cold chain system in Niger, and their contributions are important and much needed. However, many research participants working at the district or facility level felt that the partner contributions were too centralized at the national and regional level and thus did not accurately respond to the true needs at the local level. One example provided was related to the type of CCE procured for district and facility levels that has fans that easily get dusty in the environment and cause the equipment to fail if not regularly cleaned. Another example of a centralized decision is the procurement of spare parts, typically done at the central level for economies of scale yet unresponsive to the urgency of the need for the spare parts at the sub-national levels. Participants from the regional level expressed preference for managing spare parts themselves, from procurement to distribution, instead of depending on the central level.

A second finding within cold chain prioritization is that cold chain management and maintenance is not seen as an independent priority of the health system. Rather, it is closely tied to immunization activities and service delivery. This link, while logical given CCE is primarily stored for vaccines, currently translates to training that is not specific enough to address the varying maintenance tasks required of staff to perform. For example, health workers in facilities should prioritize preventive maintenance, while technicians need more technical skills and knowledge to be able to diagnose problems and replace spare parts. Participants shared that training is available yet not adequately focused on specific tasks for different staff.

#### Theme 3: knowledge sharing across the system

The final theme reflects the need for more tailored training, knowledge sharing, and clarification of roles and responsibilities across all team members who are involved in managing and maintaining CCE.

There is a discrepancy between the expectations that staff have for training and knowledge sharing, and what is actually provided to them. Regional and national level officers report that training is provided to sub-national level staff, but it may not be in the traditional seminar-style training. District level CCE technicians and immunization officers reported feeling that they do not receive adequate training on CCE maintenance. By their own admission, they often patch equipment as a quick fix based on their experience instead of a formally structured approach, such as by following a standard operating procedure. The technicians also indicated that they often rely on each other for support when faced with a problem they cannot fix, although in an ad hoc manner. While peer-to-peer support is valued and important, respondents highlighted a need for more tailored training to repair equipment. Results show that there is not a common understanding of what training is required or available for different team members at the different levels of the system, and that the preference is for a variety of training methods (seminars, mentorship, peer-to-peer support) to be available.

Limited understanding of roles and responsibilities and how different team members should collaborate results in inadequate sharing of information and inefficient use of human resources. While staff understand their individual role within the cold chain maintenance system, results show that there is not a clear connection made between the different roles and how they support each other. A concrete example of this is that the cold chain technicians do not receive temperature reports; the reports are only shared with the departmental immunization coordinator and regional immunization coordinator. Another example was a disagreement on who is responsible for preventive maintenance, the technicians or the health facility staff, each one putting the responsibility on the other. This confusion of roles and responsibilities is demotivating for staff.

### Concepts for potential solutions

During both co-creation workshops, participants responded to seven “How Might We” questions that were designed to convert problems identified through data collection and synthesis into design challenges (Table 2). Through this process, participants identified an initial 15 potential solutions to explore. Participants then aligned around four final concepts as feasible and the most impactful ideas to explore for improving the Niger cold chain maintenance system, as described here.

**Table 2:** “How Might We” questions designed for the co-creation workshop

● How might we incentivize users at the health facility level to do preventive maintenance?
● How might we more effectively utilize temperature data and reports to anticipate maintenance needs?
● How might we create an agile maintenance system that responds quickly and proactively to maintenance needs?
● How might we create a preventive maintenance system that does not depend on TPM?
● How might we create a preventive maintenance system that does not depend on TPM?
● How might we promote knowledge sharing and assistance between TPMs?
● How might we create self-sustaining, locally managed training methods at the regional, district and municipal levels?
● How might we create effective communication channels between all the actors involved in maintenance?

#### Concept 1: create an online platform to connect people involved in cold chain maintenance and to track and share knowledge across regions

This concept was designed to address the challenge of communication channels between all stakeholders, to promote knowledge sharing, and to reinforce the use of temperature data. Insights implied that structured communication between all stakeholders is necessary to ensure that the system is functioning properly and that information is being shared. It is important to create alternate systems of direct communication that are more responsive and can quickly address questions that arise.

This proposed platform would allow stakeholders to gather all information related to CCE in one place, ensuring not only good documentation but a more agile and individualized approach to accessing resources, whether in the form of technical guidance or knowledgeable people. While the participants had different ideas on the type of information that should be included on the platform, the common element was to have an easily accessible digital tool to centralize all information. The expectation with this concept is that the use of a digital platform would put the information at the fingertips of all those who need it, without any of the problems associated with traditional communication methods (email, WhatsApp, etc.).

#### Concept 2: develop different technical resources on CCE best practices and knowledge

This concept addresses knowledge sharing and the importance of creating self-sustaining, locally managed training methods. Participants agreed that the technical resources should be in a variety of material formats, such as: printed and physical materials that can be used on a daily basis for quick problem solving with visual representations; videos that can show step-by-step instructions for resolving daily and common challenges with CCE and can be shared and stored on the online platform; and a training module with theoretical and practical knowledge and activities to ensure new staff feel confident to do their job. This concept would enable access to materials that were developed within the system, providing valuable knowledge that is tailored to the specific context to support people to be able to do their job. Participants did not explore how to effectively develop and manage technical materials to ensure their utilization; this aspect would be further explored through prototyping.

#### Concept 3: use gamification and friendly competition to motivate staff to value maintenance

This concept addresses the issue of motivation to conduct preventive maintenance on CCE. This explores the idea of creating weekly challenges among health workers in districts or regions using different formats, such as quizzes on maintenance, collection of temperature data, or a task checklist to be completed. To create a friendly competition for this, scores can be shown on a dashboard on the platform periodically as designated useful by users. Participants identified this as a way to maintain frequent communication and ensure that there is no loss of information. Offline activities can include recognizing a maintenance “super star” at the end of the year to celebrate achievements, bring together all stakeholders, and recognize efforts. This type of weekly competition and annual celebration and recognition is a way to strengthen the ties between stakeholders and motivate individuals to remain committed to preventive maintenance.

#### Concept 4: develop and use a real time tracking system of maintenance activities

This concept is related to the questions of how to more effectively use temperature data and reports to anticipate maintenance needs and to create a more agile maintenance system. The concept is a system that reminds people in charge of preventive maintenance of the daily and weekly tasks required for the CCE, such as collecting temperature data, cleaning the CCE, and removing dust from the fans. This would contribute to a more structured system to guarantee that the activities and tasks to maintain the CCE are performed on time.

## Discussion

This HCD study is unique in that it engaged people directly involved and indirectly implicated in CCE management and maintenance to gain a nuanced understanding of their view of gaps in the system and included them in designing solutions to the challenges they face related to CCE management and maintenance. This study used qualitative interviews guided by an HCD approach to identify the reality of experiences, challenges, and priority needs of CCE technicians and health workers involved in using CCE. The insights highlighted contextual barriers and recurring challenges that were previously not well-understood. Viewed through a technician’s perspective, each individual has a unique set of institutional and structural circumstances that intersect and interact to enable or constrain their ability to perform their job.

While general challenges related to CCE maintenance systems in LMICs are understood broadly through some research and anecdotal evidence, this HCD study in Niger provides greater insight and understanding as to the underlying obstacles that result in the known challenges. Results validate some of the known causes of a poorly functioning maintenance system, such as inadequate and delayed financial support for maintenance that in Niger largely rests with partners. Training is often noted as lacking; however, the results revealed the need for nuanced and tailored training. This reflects a similar study on installation of new CCE in three countries that highlighted the varying effectiveness of training, insufficient information in the training, and lack of tailored information for trainee roles and skill levels [14]. As noted by participants in Niger, training should be more prioritized and not just an add-on topic within other immunization training, as it can lose the required technical details to make it effective. This issue was also revealed through the lack of clarity of roles and responsibilities, especially as it relates to preventive maintenance on CCE, which could be clarified through delineating roles and responsibilities, reinforced with more tailored training. Prioritization of cold chain management and maintenance as a key component of the health system requires dissociating it from immunization programs, particularly as it relates to training opportunities to make it easily accessible and tailored to staff.

One important and interesting insight from this study is the recognized convoluted lines of communication between team members who are involved in management and maintenance of CCE. Participants noted that TPMs do not have a direct line of communication with SERMEX. This may result in delays in repairs or lack of understanding the technical aspects of a required repair, both of which are notable challenges with maintenance systems. It is possible that this lack of communication may also contribute to the inability to plan for and procure spare parts in a timely manner as maintenance activities are not regularly reported to those who are ultimately responsible. This points to the need for a shift in collaboration and communication methods. Effective, localized collaboration is key to increase staff empowerment and expertise and to be able to respond to maintenance needs in a timely manner.

The co-creation workshop created an opportunity for stakeholders to be involved in a constructive, creative and inclusive process that encouraged contributions and ideas from different perspectives. The key insights and challenges identified during data collection and synthesis informed the overarching principles of design and the concepts developed by participants during co-creation. Often when immunization programs are planning annual budgets and workplans, activities related to maintenance include additional training and operational costs for maintenance, yet this planning falls short of the system changes that need to be made to ensure training is effective and operational costs are available when needed. Alternatively, the ideas that came out of the co-creation session were innovative ideas for the system but directly related to personalized interventions that would support technicians and immunization officers to fulfill their jobs. Further prototyping and testing is required to fine-tune the ideas to design and operationalize.

A unique aspect of this study was to conduct two co-creation workshops: one online and one in-person. Having the two workshops with different audiences allowed the solutions generated to have a global perspective but tailored to a local context. This challenged participants to think further outside of the box and create more detailed solutions. Rather than an idea of having more training, participants were able to get into the specifics of improving training challenges such as decoupling CCE training from general immunization training to offer a more information-specific approach.

It is interesting to note that digital technology was a central component of many of the concepts that were designed by participants. The government of Niger recently launched a significant effort to have innovation and technology as one of the pillars of its socio-economic plans and to encourage innovations in e-health [15]. This initiative, together with the growing access to telecommunications networks, smartphones, and digital technologies in other health areas, may have influenced the inclusion of digital health in the potential solutions. This demonstrates the participants’ ability to recognize approaches or technologies as beneficial to be able to address the identified obstacles, and to have the vision to see how they can be adapted for their own purposes.

It is notable that participants did not prioritize the issue of slow funding processes for the maintenance system during the co-creation workshop, even though government financial managers participated in the study and co-creation. According to the insights, the perceived financial responsibility for maintenance largely sits with partnering organizations, which may take away the agency of financial managers to prioritize maintenance. It would be interesting to further explore the financial management aspect to elevate its priority and identify potential solutions.

These concepts provide the basis for further exploration and prototyping of the different ideas to understand the feasibility, the level of interest, and to test out different iterations of the concepts to be most appropriate for the Niger context and the CCE maintenance system. Many of these novel ideas need to be further explored by first prioritizing ideas to prototype and test through a feasibility matrix. For example, a digital platform should be designed with the telecommunications network in mind, recognizing that not all areas have access to the network. In addition, the concept of developing tailored technical tools should include a practical approach to ensure those tools are used, acknowledging that many technical resources are not put into use or realistically adapted [16]. Policymakers and funders can support the development and implementation of these concepts into feasible solutions to support the maintenance system.

This HCD approach was used to explore options for the CCE maintenance system in Niger, yet it has implications for all global health challenges and demonstrates a methodology that can encourage key stakeholders to think about problems and solutions differently. This challenges partners to create ways to engage with not only high system level decision makers but also stakeholders whose jobs and responsibilities are influenced by those decisions. This may require more time and effort, yet the tailored solutions that can be identified may be more successful and sustainable in the long run.

### Limitations

This study had some limitations. While the sample size was small and not geographically representative of the whole country, there are likely similarities with other regions; therefore, solutions generated may address challenges experienced by other regions. Another limitation is that HCD typically continues on to prototype, develop and repeatedly test the concepts, which this effort was not able to do due to budget and time constraints.

## Conclusion

Utilization of an HCD approach revealed underlying causes of known challenges to the CCE maintenance system that were not previously well-understood in Niger or in other LMICs. Importantly, CCE technicians and health workers co-designed innovative, tailored, and responsive ideas to address challenges faced by personnel when managing and maintaining CCE. These ideas have the potential to have impact and be effective to address system challenges. These unique ideas go beyond the typical responses from governments and partners to address issues with the maintenance system.

Based on this experience, a few observations are relevant:

- HCD is a demonstrated approach to better understand underlying drivers of system challenges and could be applied productively to a range of problems.
- Insights gained from this study in Niger may be relevant to other LMICs and CCE maintenance systems.
- Donors can better target investments towards interventions that respond to the true needs of the people who work in the system.

While Niger’s immunization program is currently pursuing opportunities for funding to prototype and test the developed concepts, inclusion of national stakeholders, partners, and funders during the validation meeting elevated the importance of strengthening the maintenance system and provided insight into previously misunderstood drivers of poor maintenance.

## Data Availability

The data presented in this study are available on request from the corresponding author. The data are not publicly available due to privacy concerns of the respondents.

## Abbreviations

CDI: Departmental immunization coordinator
CCE: Cold chain equipment
CRI: Regional immunization coordinator
HCD: Human-centered design
LMIC: Low- and middle-income country
TPM: Versatile maintenance technician
SERMEX: Maintenance and repair service for operating equipment
